# A conceptual framework for contemporary professional foot care practice: ''The value based digital foot care framework''

**DOI:** 10.1186/s13047-021-00465-9

**Published:** 2021-03-25

**Authors:** Deschamps Kevin, Antoine Brabants, Chris Nester, Gabriel Gijon-Nogueron, Engin Simşek, Veronica Newton

**Affiliations:** 1 Department of Podiatry, Artevelde University of Applied Sciences, Ghent, Belgium; 2 KULeuven-Department of Rehabilitation Sciences- Musculoskeletal Rehabilitation Research Group, Campus Brugge, Spoorwegstraat 12, 8200 Brugge, Belgium; 3 Division of Podiatry, Haute Ecole Leonard De Vinci, Bruxelles, Belgium; 4 School of Health & Society,Brian Blatchford Building, Frederick Road Campus, University of Salford, M6 6PU Salford, UK; 5 Department of Nursing and Podiatry, University of Malaga, Malaga, Spain; 6 School of Physical Therapy and Rehabilitation Sciences, Dokuz Eylul University, İzmir, Turkey

**Keywords:** Podiatry practice, Framework, Value Based Health Care, Biopsychosocial-digital model

## Abstract

**Background:**

A small minority of countries around the globe have podiatry as a recognized profession, hence, there are considerable differences among these countries when it comes to the curricula, the duration of training and legislation regulating the profession. The growth in research led evidence based practice, and the emerging digital landscape of health care practice, occur alongside trends in disease and health behaviours that strongly impact on foot health. As such, the changing complex role of the podiatrist requires critical reflection on current frameworks of practice and whether they are fit for purpose. This commentary presents a conceptual framework which sets the scene for further development of concepts in a podiatry context, reflecting contemporary health care beliefs and the changing expectations of health care and society.

The proposed conceptual framework for podiatry practice utilizes the metaphor of an electronic circuit to reflect the vast and complex interconnections between factors that affect practice and professional behaviours. The framework helps in portraying and defining drivers of practice, actual practice as well potential barriers for current and future practice.

The circuit emphasis the interconnectedness/interaction of three clusters: 1) internal factors, 2) interaction factors, 3) external factors.

**Conclusion:**

Whatever promise this new framework holds, it will only be realised through conscious development of community consensus, respectful dialogue, constructive critical appraisal, and maintaining passion and focus on improving the health of people with foot related problems.

## Background

For the countries that recognise Podiatry as a profession, the duration of training and regulatory legislation varies considerably, which influences scope of practice. Clinical and research associated innovations have the potential to facilitate changes in professional boundaries and the intellectual evidence base of practice. However, there is recognition of the difficulty and barriers in translating knowledge from research findings into practice, education, discussion and culture [1].

Currently, podiatry belongs to a health practice community facing a digital revolution, and health care providers seek to embrace digital health solutions, reshaping the health professional-patient relationship and the context within which care is provided [2, 3].

The growth in research led evidence based practice, and the emerging digital landscape of health care practice, occur alongside trends in disease and health behaviours that strongly impact on foot health. Diabetes, obesity, active lifestyles and living longer combine to mean the public health role of the podiatrist (or equivalent foot health practitioner) within society has evolved significantly.

As such, the changing complex role of the podiatrist requires critical reflection on current frameworks of practice and whether they are fit for purpose. We propose that now is a timely opportunity to revisit the frameworks that underpin Podiatry and foot health practice. We believe that there is currently a lack of conceptual frameworks providing a suitable lens through which the vast range of factors impacting on practice can be considered. We believe there is a need to consider the dynamic nature of knowledge and practice to represent evolving approaches.

The objective of this commentary is to present a conceptual framework, namely the Value Based Digital Foot Care Framework, for use by podiatry students, lecturers, therapists, scientists and policymakers. The term framework includes a number of concepts, theories and empirical findings from the (scientific) literature and is used to show relationships and stimulate innovation among these components. It defines a way of explaining phenomena and serve as basis for dealing with complexities.

Here, it will set the scene for further development of concepts in a podiatry context, reflecting contemporary health care beliefs and the changing expectations of health care and society. In offering this perspective we hope to invite a significant acceleration in the maturity, intellectual quality, and dynanism that drives Podiatry and foot health care practice forward.

### The conceptual framework

**Fig. 1:**
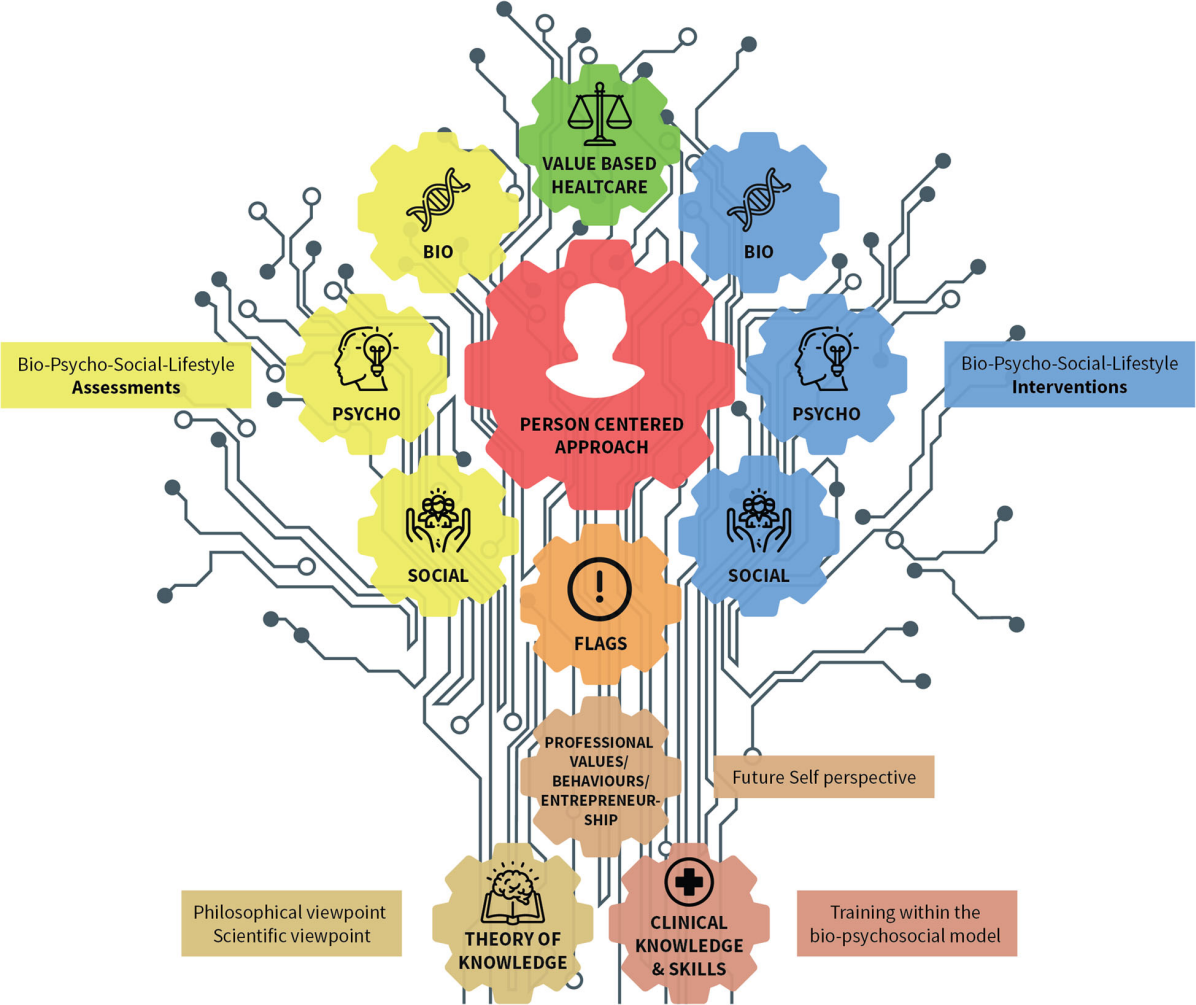
Illustration of the Value Based Digital Foot Care Framework

The proposed framework for podiatry practice utilizes the metaphor of an electronic circuit to reflect the vast and complex interconnections between factors that affect practice and professional behaviours (Fig. 1). The circuit emphasis the interconnectedness/interaction of numerous linked concepts, models, paradigms, theories and practices which have been classified into three clusters. The framework helps in portraying and defining drivers of practice, actual practice as well potential barriers for current and future practice.

The infographic (Fig. 1) illustrating the conceptual framework has been created since it captures the different components of the framework nicely and in an easy way to remember and apply. Two examples about how this framework could be used include: (1) as a tool when critically reflecting about a future self perspective when dealing with under-graduate students, (2) as a tool to develop a value based healtcare pathway in a certain foot related condition or disease.

### Cluster 1: internal factors

The Podiatrist is represented at the base of the circuit and the infographic focuses on internal factors using a person centred approach. Internal factors include past experiences, perceived complexity of a health care and/or support question and expertise of the podiatrist. Throughout their learning, a podiatrist develops requisite knowledge and skills within the boundaries of national legislation. These are often classified within competency frameworks which align with professional values and behaviours [4, 5]. As they learn and develop, student podiatrists are encouraged to embrace the principles of evidence based practice and a person centred approach. The term person centred approach is used specifically here in order encourage podiatrists to adopt a patient care perspective that is beyond the condition and tailored to the individual/patients’ wants and needs. In addition, learners, should be encouraged to develop knowledge by many ways of knowing: emotion, faith, imagination, intuition, language, memory, reasons, sense perception) [6, 7]. Encouraging this approach, allows learners to appreciate how sociological, technological, and political change, can influence their future-self perspective and clinical practice [8].

### Cluster 2: interaction factors

Presented as the central section of the circuit, interaction factors focuses and define the interplay between the podiatrist and the person. Critical appraisal of current podiatric practice and published research reveals that the primary focus has always been the physical determinants of foot related complaints [9]. This biophysical approach may be appropriate for some pathologies, however, research suggests a strong influence of psychological and social factors in many musculoskeletal conditions [10–14] reflecting non biophysical elements. Therefore, this may warrant review of current podiatry education and practice, with training and research into the assessment of mental health, somatising traits and trends, health beliefs and behaviours [15]. By doing so we invite enhancement of practice, and rejection of single factor thinking that limits the scope of practice and thereby outcomes for patients.

In the UK, Standards of Proficiency (HCPC - add EU ) state podiatrists should be able to engage in effective and professional interactions and adopt a person-centered approach. This approach shapes the quality of the patients experience and is important where complex interactions are required to support maintenance of foot health. In current healthcare, multidimensional interventions and person centred self care has become the gold standard. Therefore, it is appropriate podiatrists employ methods to identify how multidimensional approaches are applied, for example use of clinical (red flags) and psychosocial flags (yellow, blue and black flags) that affect outcomes for patients [16].

### Cluster 3: external factors

This framework stresses a biopsychosocial approach, in which individuals’ needs and wants are respected and value is placed on a patient as the expert in their own life and health. This moves away from the model of what is “done” by the professional, towards the value added for the patient. A person-centered approach aligns with the belief that health care systems must be equitable, sustainable and use resource in a transparent way. There is already a distinct shift towards a ‘value-based healthcare (VBH)’ delivery model, where providers are paid based on patient health outcomes rather than the fee-for-a service approach, which pays for what is delivered, regardless of outcome [17, 18]. Despite VBH being advocated as an optimal model for future health care systems, the solidarity between the professional and patient, deeply rooted in this model poses significant challenges to health care providers. These challenges, together with opportunities and constraints within society and influenced by governments further highlights the need for a flexible and innovative podiatrist who can effectively include patient outcomes in their care plans and management framework.

### The Digital component

The last element represented in the conceptual framework is the ‘digital component’ which is visualized by the electronic circuit. This was chosen not only to illustrate the interconnectedness/interaction of the three clusters and their components, but also the emergence of ‘digital health’. The latter is an advancing phenomenon in society independent of modern health care systems and hence is no longer an optional aspect of the care a person receives. Societal expectations associated with digital experiences ask that health professionals and patients/clients integrate and use digital technologies in flexible ways [3, 19].

We therefore wonder, whether a,’biopsychosocial-digital’ approach to foot related problems and needs is appropriate. Digital skills and behaviours must, we believe, be embedded in all curricula and thus at the start of the journey of professional learning, as they will hereafter be central to almost all aspects of health professional life. Indeed, we have all experienced the transformational impact of digital care on health matters in the ongoing 2019-nCoV outbreak, whether it be public health messages, digital notifications of test outcomes, tracking technologies, or Podiatry care through video and voice based consultations.

## Conclusions

Whatever promise this new framework holds, it will only be realised through conscious development of community consensus, respectful dialogue, constructive critical appraisal, and maintaining passion and focus on improving the health patients of with foot related problems. Indeed, this would be evidence of a healthy professional framework in action and purposefully evolving the professions to meet changing societal needs.

## Data Availability

Not applicable.
