# Correction to: A conceptual framework for contemporary professional foot care practice: “The value based digital foot care”

**DOI:** 10.1186/s13047-021-00473-9

**Published:** 2021-04-08

**Authors:** Kevin Deschamps, Antoine Brabants, Chris Nester, Gabriel Gijon-Nogueron, Engin Simşek, Veronica Newton

**Affiliations:** 1 Department of Podiatry, Artevelde University of Applied Sciences, Ghent, Belgium; 2 KULeuven-Department of Rehabilitation Sciences, Musculoskeletal Rehabilitation Research Group, Campus Brugge, Spoorwegstraat 12, 8200 Brugge, Belgium; 3 Division of Podiatry, Haute Ecole Leonard De Vinci, Bruxelles, Belgium; 4 School of Health & Society,Brian Blatchford Building, Frederick Road Campus, University of Salford, Salford, M6 6PU UK; 5 Department of Nursing and Podiatry, University of Malaga, Malaga, Spain; 6 School of Physical Therapy and Rehabilitation Sciences, Dokuz Eylul University, İzmir, Turkey

**Correction to: J Foot Ankle Res 14, 22 (2021)**

**https://doi.org/10.1186/s13047-021-00465-9**

Following the publication of the original article [1], we were notified that the first and last names of the first author were switched.

The original article has been corrected.
